# Rat Bite Fever Resembling Rheumatoid Arthritis

**DOI:** 10.1155/2016/7270413

**Published:** 2016-04-12

**Authors:** Ripa Akter, Paul Boland, Peter Daley, Proton Rahman, Nayef Al Ghanim

**Affiliations:** ^1^Department of Internal Medicine, Memorial University, St. John's, NL, Canada A1B 3V6; ^2^Disciplines of Medicine and Laboratory Medicine, Memorial University, St. John's, NL, Canada A1B 3V6; ^3^Department of Medicine, Memorial University, St. John's, NL, Canada A1B 3V6; ^4^Eastern Health, St. John's, NL, Canada A1C 5B8

## Abstract

Rat bite fever is rare in Western countries. It can be very difficult to diagnose as blood cultures are typically negative and a history of rodent exposure is often missed. Unless a high index of suspicion is maintained, the associated polyarthritis can be mistaken for rheumatoid arthritis. We report a case of culture-positive rat bite fever in a 46-year-old female presenting with fever and polyarthritis. The clinical presentation mimicked rheumatoid arthritis. Infection was complicated by discitis, a rare manifestation. We discuss the diagnosis and management of this rare zoonotic infection. We also review nine reported cases of rat bite fever, all of which had an initial presumptive diagnosis of a rheumatological disorder. Rat bite fever is a potentially curable infection but can have a lethal course if left untreated.

## 1. Introduction

Rat bite fever (RBF) is a systemic febrile illness caused by either* Streptobacillus moniliformis*, common in Western countries, or* Spirillum minus*, which is the most prevalent pathogen in Asia [1, 2]. It is transmitted to humans by bites or scratches from infected rats. Classic clinical features include fever, rash, and polyarthritis [1]. When RBF presents with symmetrical polyarticular synovitis, rheumatoid arthritis may initially be diagnosed incorrectly, leading to delay in appropriate therapy [3–7]. Complications of RBF include septic arthritis, endocarditis, and rarely discitis, as in our patient. The mortality rate of untreated cases ranges from 7% to 13% and for cases complicated by endocarditis it can be up to 53% [1, 2].

## 2. Case Report

A 46-year-old female was admitted with a one-week history of fever and symmetric polyarthritis of the distal upper and lower extremities, with thirty minutes of morning stiffness. A few days prior to her admission, she had a one-day history of nausea, vomiting, and diarrhea. She denied recent travel or illicit drug use. Her previous medical history was significant for a seizure disorder, irritable bowel syndrome, chronic mechanical back pain, and iron deficiency anemia. Her family history was unremarkable for any rheumatological illness.

On examination, she was febrile (38°C), tachycardic (130 beats per minute), and hypotensive (96/64 mmHg). The most prominent physical finding was effusions in her wrists, ankles, and selected metatarsophalangeal joints. Her cardiopulmonary, abdominal, and dermatological examinations were otherwise unremarkable. Erythrocyte sedimentation rate was 76 mm/hr (normal: 0–12 mm/hr) and C-reactive protein was 149 mg/L (normal: 0–8 mg/L). There was a mild leukocytosis of 11.1 × 10^9^/L (normal: 4.8–10.8 × 10^9^/L). Initial blood culture and serological tests including hepatitis B and hepatitis C, parvovirus B19, HIV, Lyme disease,* Chlamydia trachomatis*, and* Neisseria gonorrhea* were negative. Rheumatological workup including rheumatoid factor, anti-nuclear antibody, anti-cyclic citrullinated peptide antibody, anti-neutrophil cytoplasmic antibodies, anti-dsDNA antibody, and complement levels was all within normal limits. Chikungunya virus serology was not ordered as this diagnosis was unlikely given she had not travelled. A presumptive diagnosis of seronegative rheumatoid arthritis was made, based on the clinical presentation of symmetrical inflammatory polyarthritis and negative infectious workup. She was started on a trial of oral prednisone. She experienced mild improvement in her synovitis. She was discharged home on triple therapy for rheumatoid arthritis which included methotrexate, sulfasalazine, and hydroxychloroquine.

**Figure 1:**
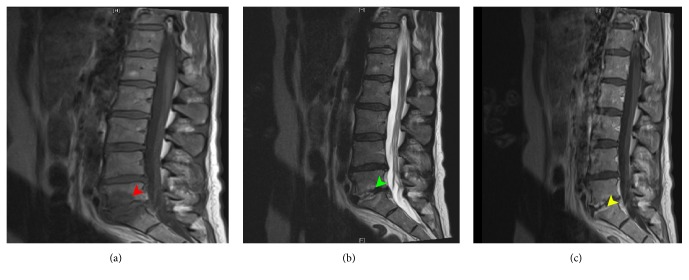
Sagittal MRI pulse sequences of lumbosacral spine at presentation. (a) (T1-weighted) shows markedly reduced signal at the L5-S1 level while (b) (T2-weighted) shows increased T2 signal both in keeping with edema. (c) shows enhancement of the vertebral end plates. All findings are in keeping with discitis.

The patient returned to the hospital next day with worsening synovitis, fever (39°C), and new onset of back pain localized to the lumbar spine. Sulfasalazine and methotrexate were discontinued because of a new transaminitis (aspartate aminotransferase 105 U/L (normal: 0–37 U/L); alanine aminotransferase 114 U/L (normal: 0–55 U/L)). The ESR was elevated at 124 MM/HR and C-reactive protein at 170 mg/L. Right ankle aspiration was performed followed by methylprednisolone injection due to ongoing severe pain. The synovial fluid sample was inadequate for gram stain; however, the culture was negative. She then received intravenous methylprednisolone, 250 mg every 24 hours for 2 days without improvement. Repeated blood culture grew* Streptobacillus moniliformis* in the anaerobic flask. MRI revealed L5-S1 discitis (Figure 1) and transthoracic echocardiogram showed no evidence of endocarditis. On further questioning, the patient admitted to having a pet rat and a pet cat, both of which had died of an unknown illness in the week prior to the initial presentation to hospital. The patient was told by a local veterinarian that the rat was “in kidney failure” though further details are unavailable. The patient spent the night prior to the death of the rat comforting the ailing animal in her arms. During this time, she received a scratch to her chest.

A diagnosis of RBF was made. The patient then was treated with intravenous ceftriaxone with discontinuation of steroids and hydroxychloroquine with symptomatic improvement. She was discharged home with a 3-month course of intravenous ceftriaxone on the advice of infectious disease and neurosurgery specialists to ensure resolution of her discitis. Three months after discharge, the patient was well with complete resolution of her arthritis, marked improvement in the lower back pain, and normal inflammatory markers. A repeat MRI showed resolution of the discitis.

## 3. Discussion

*Streptobacillus moniliformis* is not routinely reported to public health authorities in most jurisdictions, and hence the true incidence rate is unknown. We report a challenging case of RBF with discitis involving L5-S1, which was initially presumed to be rheumatoid arthritis. RBF with discitis is extremely rare. To our knowledge, this is the third reported case of discitis associated with rat bite fever. Dubois et al. reported a case of RBF with spondylodiscitis involving T5-T6 and L2-L3 [12]. Nei et al. described another case of discitis involving L3-L4 [13].

Apart from direct rat bite or scratch, infection can also spread to humans by bites or scratches from animals that prey on rodents, such as cats, dogs, and pigs [8].* Streptobacillus moniliformis* is part of the normal nasopharyngeal flora of rats. Other rodents such as mice, guinea pigs, ferrets, squirrels, and gerbils also colonize this bacteria [7]. Ingesting contaminated food products can also cause RBF, as described in Haverhill, Massachusetts, in 1926 [8]. RBF in farmers due to ingestion of unpasteurized milk has been reported [8]. Pet owners, children, and those working in pet shops and animal research laboratories are at an elevated risk of contracting this infection [14]. Ninety percent of patients develop fever within 3–10 days of exposure, which can follow a relapsing pattern [2]. Typically a maculopapular, petechial, or purpuric rash is seen in the extremities and biopsy is consistent with a leukocytoclastic vasculitis [2, 15, 16]. Other symptoms include vomiting and headache [14]. A migratory polyarthritis is seen commonly affecting the hands, wrists, elbows, knees, and, rarely, the sternoclavicular and sacroiliac joints [2, 3, 17, 18].* Streptobacillus moniliformis* septic monoarthritis is described, in some cases requiring surgical debridement [19, 20]. Additional complications include osteomyelitis, pericardial effusion, endocarditis, pneumonia, meningitis, and multiorgan failure [1, 2, 14, 20].

The pathogenesis of arthritis in RBF is multifactorial. Systemic symptoms, such as fever and rash, may occur with a sterile synovial fluid culture, suggesting a reactive phenomenon due to an immune mediated process. In other cases, synovial fluid cultures are positive with or without bacteremia suggesting a direct infectious process [4, 21, 22]. Features that suggest an immune mediated phenomenon may include vasculitic rash, hypocomplementemia, and cryoglobulinemia [23]. Wang and Wong suggest that septic arthritis caused by* Streptobacillus moniliformis* detected in synovial fluid without bacteremia is a separate entity with distinct clinical features in which fever and rash are uncommon [21].

The diagnosis of RBF can be challenging as blood cultures are usually negative [14].* Streptobacillus moniliformis* is a facultatively anaerobic, highly pleomorphic gram negative bacillus [21]. Bacteria can vary in length from two to fifteen *μ*m. Its growth can be inhibited by sodium polyanethol sulfonate, an anticoagulant found on most aerobic culture bottles [21]. Therefore, this organism is more likely to grow in anaerobic cultures [3]. Positive blood, synovial fluid, or rarely skin lesion culture followed by identification using gas chromatography or sequencing of 16 s rRNA genes can confirm the diagnosis [3–6, 16]. Up to 25% of affected patients may have a false positive serology test for syphilis [23].

Although this infection is difficult to diagnose, its prognosis is favorable. The standard treatment of RBF is penicillin or, in the case of penicillin allergy, tetracycline [21].* Streptobacillus moniliformis* is also susceptible to cephalosporins, carbapenems, erythromycin, and clindamycin [21].

Table 1 summarizes nine cases of RBF mimicking a rheumatological disorder. Six out of the nine cases received steroid therapy (Table 1). In a case described by Tattersall and Bourne, a patient received cyclophosphamide when inflammatory vasculitis was suspected (Table 1). These cases highlight the importance of maintaining a broad differential that includes RBF when assessing potential cases of rheumatoid arthritis. The positive blood culture was the main clue to the diagnosis in our case. This case report also highlights the potential hazard of misdiagnosis and treatment with immunosuppressive agents. Infectious etiology is always on the differential, such that a zoonotic exposure history and blood cultures should be obtained when assessing a patient with fever and arthritis. Also occupational, travel, and recreational history should be sought for potential rodent exposure in suspected cases.

## Figures and Tables

**Table 1:** Reported Cases of Rat bite fever with initial presumed diagnosis of rheumatological disorders.

Study/year/ [reference]	Age/sex	Rat bite/scratch	Occupation	Family history of rheumatological disorders	Clinical features	Affected joints	Joint aspirate analysis	Joint aspirate culture	Identification method of *Streptobacillus moniliformis*	Blood culture	Rheumatological workup	Joint erosion	Initial presumed diagnosis	Treatment	Outcome
Legout et al./2005 [3]	60/female	Rat bite	Pet shop employee	Father- seropositive rheumatoid arthritis	Fever and polyarthritis	Symmetrical affecting small joints of both hands and ankles and right knee	Right knee synovial fluid: leukocytosis (40 × 10^9^/L) with 90% neutrophils	GNB	PCR amplification of part of 16S RNA gene	Negative	RF, ANA, ANCAs, specific anti-filaggrin antibody, and cryoglobulin were negative	No erosion	Rheumatoid arthritis	*Initial*: NSAIDs and IV methylprednisolone 500 mg/day for 3 days, no improvement *Postculture*: arthrotomy of right knee and 4 weeks of antibiotics which included IV penicillin followed by oral rifampin and clindamycin	Successfully treated

Dendle et al./2006 [4]	49/female	Rat bite	Not reported	Not reported	Polyarthritis, fever, rash, pneumonia, and hepatitis	MCP, wrists, knees, right elbow, and right ankle	Right elbow: numerous PMN No formal analysis-sample clotted	Pleomorphic GNB	16S rRNA gene sequencing	Negative	ANA and RF compliment levels were normal	No erosion	Rheumatoid arthritis or Still's disease	*Initial*: oral prednisone 25 mg daily with worsening synovitis *Postculture*: doxycycline 100 mg twice daily for 6 weeks	Successfully treated

Stehle et al./2003 [5]	72/male	Rat bite	Not reported	Not reported	Polyarthritis	Both knees, elbows, and left 3rd MCP	Right Knee: leukocytosis (around 50 × 10^9^/L) with 83% neutrophils Rearthrocentesis of both knees, right elbow, and left 3rd MCP: analysis not reported	*Streptobacillus moniliformis* grew on repeat synovial fluid culture	16S rRNA gene sequencing	Negative	Not reported	No erosion	Atypical rheumatoid arthritis	*Outpatient*: NSAID and deflazacort for almost 1 month, no improvement *Postadmission*: bolus of IV steroids, minimal improvement *Postculture*: broad spectrum antibiotics	Successfully treated

Holroyd et al./1988 [6]	59/male	No	Not reported	Not reported	Fever and polyarthritis	PIP, MCP, wrist and knees, ankles, elbows, and shoulders bilaterally	Left knee: leukocyte 3,700/mm^3^ with 80% PMN Left wrist: 57,000/mm^3^ and 90% PMN	Left wrist: pleomorphic GNB with bullous swelling	Gas chromatography of the cellular fatty acid of organism	*Streptobacillus moniliformis*	Negative RF and weakly positive ANA 1 : 40	Not reported	Rheumatoid arthritis	*Outpatient*: patient took NSAIDs for 1 day prior to admission *Postculture*: ticarcillin and gentamicin; penicillin G for total 10 days	Successfully treated

Kanechorn and Niumpradit/ 2005 [7]	61/female	Rodent bite	Retired nurse	Not reported	Fever, petechial rash, myalgia, and symmetrical polyarthritis	Fingers, wrists, knees, and ankles	Site of joint aspiration not reported. Analysis: leukocyte counts of over 64,000 cells/mm^3^ and all neutrophils	Negative	Not reported	Negative	ANA and RF negative	Not reported	Septic arthritis and rheumatoid arthritis	*Initial*: erythromycin, Ibuprofen as well as rabies vaccination and tetanus toxoid prior to admission *Postadmission*: dexamethasone 4 mg every 6 hours, amoxicillin/clavulanic acid plus doxycycline, no improvement *After joint analysis*: ceftriaxone and penicillin G for 4 weeks, arthrotomy and debridement of joints, unreported sites of joints	Successfully treated

Abdulaziz et al./2006 [8]	68/male	Rat exposure, no bite	Dairy farmer	Not reported	Symmetrical polyarthritis, rash, fever, myalgias, and headache	PIP's, MCP's, wrists, ankles, and knees	Left knee: white blood cell count of 19,250/mm^3^, 84% PMN leukocytes, and CPPD crystals	Negative	Not reported	Pleomorphic gram negative bacilli	Not reported	No erosion	Acute polyarticular pseudo gout	*Initial*: ibuprofen and NSAIDs *Postculture*: penicillin G for 14 days successfully treated	Successfully treated

Tattersall and Bourne/2003 [9]	56/male	Rat bite	Not reported	Not reported	Fever, rash, asymmetric polyarthritis, hand ischemia, sore throat, and loose stools	Right elbow, wrist, shoulder, left thumb MCP joint, both midtarsal joints, and right ankle	Left thumb MCP: analysis not reported Left ankle: urate crystals	Gram negative pleomorphic coccobacillus *Streptobacillus moniliformis*	DNA sequencing	Negative	Autoantibodies and ANCAs were negative	Not reported	Vasculitis or reactive arthritis	*Initial*: IV methylprednisolone and cyclophosphamide for few days with minimal improvement *Postculture*: oral doxycycline for 6 weeks	Successfully treated

Dworkin et al./2010 [10]	59/male	Rat exposure, no bite	Not reported	Not reported	Polyarthritis, diarrhea, malaise, and presumed endocarditis	knees, ankles, wrists, right elbow	Left knee: analysis not reported	Pleomorphic GNB	16S rRNA gene sequencing	Negative	ANA elevated 1 : 160 and normal compliment, RF and, ANCA levels	Not reported	Polyarthritis of infectious or collagen vascular disease etiology	*Initial*: NSAIDs and steroids *Postculture*: penicillin, doxycycline, and gentamycin for 6 weeks	Successfully treated

Budair et al./2014 [11]	29/male	Rat exposure	Manual laborer in a warehouse	Not reported	Malaise, fever, sore throat, rash, and polyarthralgia	Right second MCP, right elbow, right knee and both ankles	Right ankle aspiration: yellow cloudy fluid Analysis not reported	Culture negative	16S rRNA PCR identified organism	Negative	ANA, double stranded DNA antibody, glomerular basement membrane antibody, myeloperoxidase antibody and proteinase-3 antibodies, RF, and immunoglobulins were all normal	Not reported	Vasculitis	*Postorganism identification*: intravenous benzylpenicillin and 3 weeks of oral amoxicillin	Successfully treated

GNB: gram negative bacilli; PIP: Proximal Interphalangeal; MCP: metacarpophalangeal; RF: rheumatoid factor, ANA: anti-nuclear antibody, ANCA: anti-neutrophil cytoplasmic antibody; NSAID: nonsteroidal anti-inflammatory drug; IV: Intravenous; PCR: polymerase chain reaction; PMN: Polymorphonuclear; CPPD: calcium pyrophosphate dihydrate.

## Additional Points

- Rat bite fever is uncommon and very difficult to diagnose.
- A history of zoonotic exposure is key to diagnosis.
- Clinicians should include rat bite fever in the differential diagnosis of symmetrical inflammatory polyarthritis.
- Prognosis is good when treated appropriately but potentially lethal if left untreated.
- Repeating joint aspiration and blood cultures could increase the likelihood of a positive identification of pathogens associated with RBF.
